# Meta-analysis of the effect of static computer-assisted dental implantation on the accuracy of dental implantation in esthetic area

**DOI:** 10.4314/ahs.v25i2.40

**Published:** 2025-06

**Authors:** Zhongping Yin, Huifang Kuang, Li Li, Xi Si

**Affiliations:** 1 Center of Stomatology, The Second Affiliated Hospital of Hainan Medical University, Haikou, China; 2 Department of Stomatology, First Affiliated Hospital of Hainan Medical University, Haikou, China; 3 Department of Nephrology, Geriatric Hospital of Hainan, Haikou, China; 4 School of Stomatology, Hainan Medical University, Haikou, China

**Keywords:** Static guide plate, Dynamic navigation, Anterior teeth aesthetic district, Implant, Meta-analysis

## Abstract

**Background:**

This system evaluates the effect of static navigation systems on accuracy (platform, apical and angular deviation) in clinical studies of implant surgery in the anterior aesthetic zone to inform clinical decision-making.

**Methodology:**

Retrieve Pubmed, Embase, Cochrane library databases, as well as randomized controlled trials (RCT) and controlled clinical trials (CCT) comparing dynamic navigation and static guidance published by the US Clinical Trial Registry before May 1, 2023. The outcome indicators included in the study include measuring the deviation between the preoperative design of the implant and the actual site at the top, apex, and angle. The calculation of meta-analysis was performed using Review Manager version 5.2 software.

**Results:**

1134 articles were screened and retrieved, and finally 4 studies were included for quantitative analysis. Meta analysis results showed that the two groups had lower depth angle deviation (WMD=-0.30, 95%CI: (0.24, 1.78), P=0.01) at the top (WMD=0.14, 95% CI: (-0.26, 0.55), P=0.49) and root tip (WMD=0.25, 95%CI: (-0.34, 0.84), P=0.41) compared to the static guide plate group, and the difference was statistically significant.

**Conclusion:**

The static guide plate provides a small implant placement error, which is comparable to the error obtained using dynamic navigation systems, but dynamic navigation exhibits a smaller angle deviation.

## Introduction

Over the past thirty years, the field of implantology has significantly evolved, focusing on the aesthetic and functional benefits of prosthetic-driven implants. This evolution underscores the importance of precise implant placement for not only achieving clinically acceptable results but also ensuring high implant survival rates and long-term stability of the surrounding tissue^1^,^2^.

The advent of computer-assisted technologies has revolutionized implant surgery. Currently, two main methodologies are prevalent: static computer-assisted implant surgery (sCAIS) and dynamic computer-assisted implant surgery (dCAIS). sCAIS utilizes preoperative cone beam CT scans for planning and employs a surgical guide plate for execution^3^. Conversely, dCAIS involves real-time navigation during the procedure, allowing surgeons to adapt to intraoperative conditions^4^. These advancements have marked a significant leap in surgical precision and outcomes.

Computer-assisted implant surgery technologies are expected to meet certain standards to ensure precision, safety, and effectiveness. These standards typically include accurate preoperative planning, precise implant placement, minimal invasiveness, and compatibility with various anatomical conditions. Some technologies may not meet these standards due to limitations in their design, such as inadequate imaging resolution, lack of real-time navigation, or insufficient adaptability to different oral conditions. Additionally, the complexity of the technology and the required training for its effective use can also be factors that make some systems less suitable for certain clinical settings. This variance in meeting standards underscores the importance of continual development and evaluation of these technologies.

However, despite the progress, there remain gaps in research, particularly in the comparison of these technologies. Previous systematic reviews have focused on the accuracy of sCAIS, but often included model-based studies, lacked recent research, or did not provide a comparative analysis between dynamic navigation, static guidance, and conventional freehand methods^5^. Furthermore, there is an absence of focused analysis on patients with single missing teeth in the anterior aesthetic zone, a scenario where the precision of implant placement is critical^6^,^7^. Therefore, this review aims to fill these gaps by assessing the accuracy of static guides in such surgeries and comparing them with dynamic navigation, to explore if there is a significant difference in accuracy between these methods.

## Materials and Methods

### Retrieval Strategy

**Figure 1:**
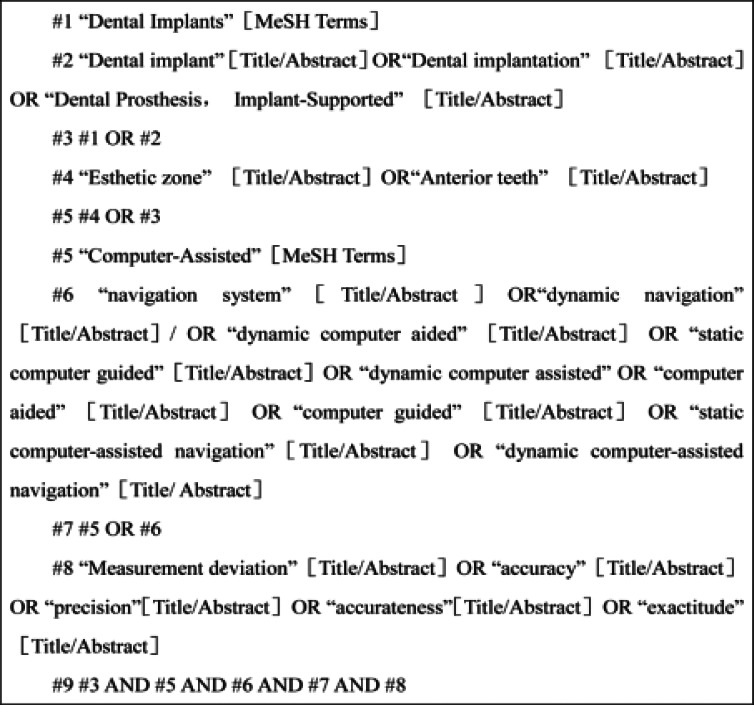
PubMed search strategy

The literature search was conducted in May 2023 in three databases (Pubmed, Embase, Cochrane library databases), with indexing terms related to implantation, computer-aided implant surgery, dynamic guidance, static guide plate, and dynamic navigation. The main MesH used in the search is as follows: “Computer assisted implant surgery+measurement bias”, with no restrictions on publication language or date. This occurs when the measurement method or instrument consistently over- or underestimates the true value of what it's measuring. In medical research, including studies on computer-assisted implant surgery, recognizing and mitigating measurement bias is crucial to ensure the validity and reliability of the results. This type of bias can significantly affect the outcomes and conclusions of a study, making it a critical factor to consider in research design and analysis. The search strategy used in PubMed is shown in Figure 1, and the search was updated in May 2023.

### Criteria for Inclusion and Exclusion of Literature

Inclusive criteria: 1. The types of studies were randomized controlled trial, quasi experimental studies and cohort study; 2. The research subjects are patients who undergo implant surgery using computer-aided implant surgery technology, without limiting their gender, age, ethnicity, nationality, etiology, underlying diseases, etc; 3. The intervention group underwent implant surgery using static guidance plates, while the control group underwent implant surgery using dynamic navigation or static guidance plates; 4. Outcome indicators: Measurement deviation at the top of the implant (mm), measurement deviation at the root tip (mm), and measurement deviation at the angle (°), including one or more of them.

Exclusion criteria: 1. Non RCT, CCT studies; 2. Repeated publications, conference papers, and poor quality literature; 3. The full text cannot be obtained through various means; 4. Animal experiments; 5. Those who perform implant surgery using computer-aided implant surgery technology that does not meet the standards; 6. Unable to obtain original information or incomplete or unusable literature reports, and unable to contact the author.

### Literature Screening and Data Extraction

All relevant literature in the preliminary search was extracted and sorted out according to the above inclusion and exclusion criteria, and researchers who had received training in evidence-based methods independently screened the literature, extracted data, and cross-checked according to the inclusion and exclusion criteria of the literature, and if there was a difference of opinion, they reached an agreement through discussion or assisted in adjudication with another researcher. Including: 1. Basic information of the included studies; 2. Details of the intervention; 3. The characteristics of the base line of the research object; 4. Required outcome indicators and their measurement data; 5. Key elements of risk of bias assessment. All the literature that meets the above criteria is repeatedly screened out, and those who do not meet the criteria are eliminated after the second screening, and finally the research literature that meets the inclusion criteria is counted.

### Literature Quality Evaluation

Use the bias risk assessment tool recommended in Cochrane Evaluation Manual 5.1.0 to evaluate the quality of Randomized controlled trial^8^. The evaluation content includes the generation of random sequence, the hiding of allocation scheme, the blinding of research objects and interveners, the blinding of evaluators, the integrity of the result data, the selective reporting of results and other biased items. The researchers make a judgment of “low risk bias” and “high risk bias unclear” for each item. If the research fully meets the above criteria, the likelihood of various biases occurring is low, with a quality level of A; if partially met, the likelihood of bias occurring is moderate, with a quality level of B; if not completely met, the likelihood of bias occurring is high, with a quality level of C.

### Statistical analysis

Meta analysis was conducted using RevMan 5.3.5 software. Heterogeneity evaluation of the literature included in the study was conducted using I2 statistics and Chi2. If P<0.10 or I2>50% was tested by Chi2, it indicates significant heterogeneity. In this case, a random effects model was used for consolidation analysis, otherwise a fixed effects model was used. The combined statistics of continuous numerical variables were evaluated using weighted mean difference (WMD) and their 95% confidence interval (CI) was calculated. P<0.05 indicates a statistically significant difference. To ensure the stability of the results, sensitivity analysis was conducted by excluding individual studies and reanalysis. In addition, the degree of publication bias was evaluated by observing the funnel plot.

## Results

### Document Retrieval Results

**Figure 2:**
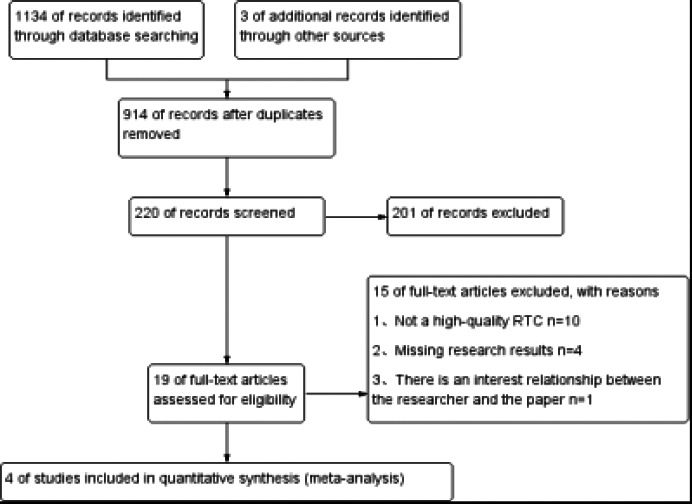
Flow chart of literature screening

A preliminary search yielded 1134 articles, and 3 related articles were supplemented through other means, totaling 1137. After removing duplicate literature through End-Note X9 software, 220 articles were obtained. After reading the title, abstract, and full text, 215 articles that did not meet the inclusion criteria were excluded, and finally 4 articles were included^9^-^12^. EndNote X9 is known for its ability to handle large volumes of data and its sophisticated filtering algorithms, which can accurately identify and eliminate duplicate entries. This ensures a more streamlined and accurate literature review process, reducing the risk of including redundant studies in the analysis. The screening process and research overview of the included articles are shown in Figure 2 and Table 1.

**Table 1:** Basic characteristics of included literature (N=4)

Study	Year	Study design	Sex(F/M)	Age	Number of implants s-CAIS/d-CAIS	Type of tooth loss	Navigation system s-CAIS/d-CAIS	Registration method
Miaozhen Wang^9^	2022	RCT	NR	NR	10/10	Single/multiple anterior tooth defects	NR	Registration stent
Dong Wu^10^	2022	RCT	22/32	19-67	57/38	Single/multiple anterior tooth defects	NR	U-type tube
Quan Liu^11^	2022	CCT	13/19	27-39	20/18	Single/multiple anterior tooth defects	coDiagnostiX	NR
Yuzhang Feng^12^	2022	RCT	18/22	23-46	20/20	Single/multiple anterior tooth defects	BLT Straumann/Nobel Active	Registration stent

**Figure 3:**
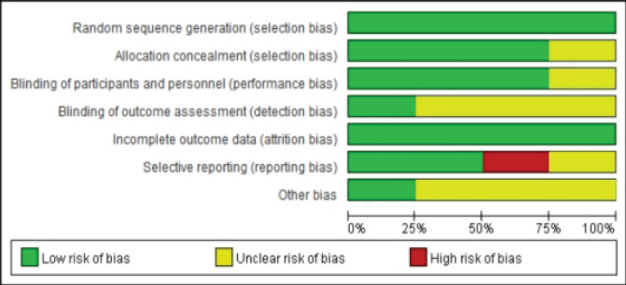
Summary of risk bias

**Figure 4:**
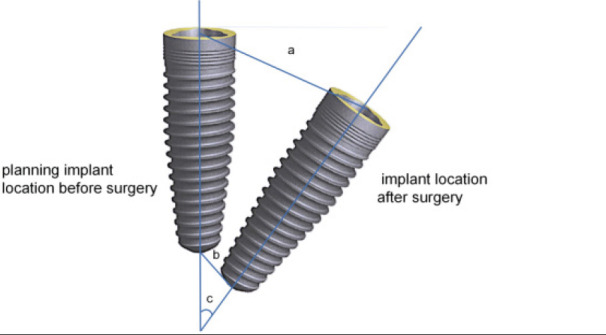
Schematic diagram of outcome indicators for the study. a: Measurement deviation at the top (mm) b: Measurement deviation of root tip (mm) c: Measurement deviation of angle (mm)

The risk of bias was evaluated using the evaluation table recommended by the Co Crane evaluator manual for the literature included in the study. All included studies were of high quality, and implementation bias, measurement bias, and follow-up bias all had lower bias risks (Figure 3). This assessment was conducted using the bias risk assessment tool recommended in the Cochrane Evaluation Manual. The evaluation focused on several criteria, including the generation of random sequences, allocation concealment, blinding of participants and personnel, blinding of outcome assessment, completeness of outcome data, selective reporting, and other sources of bias. The basic characteristics of the included study, the schematic diagram of outcome indicators, and the results of bias risk assessment are shown in Figure 4.

### Meta Analysis Results

#### Measurement Deviation at the Top of the Implant

**Figure 5:**
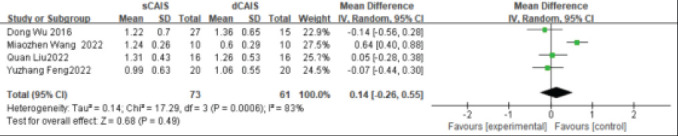
Forest plot of measurement deviation of top of implant

The measurement deviation at the top of the implant was included in 4 studies. The fixed effects model analysis results showed that there was no statistically significant difference in the measurement deviation (mm) between the preoperative design and actual implantation site of the dynamic navigation and static guide plate groups at the top WMD=0.14, 95% CI: (-0.26, 0.55), P=0.49]. (Figure 5)

#### Measurement Deviation of the Root Tip of the Implant

**Figure 6:**
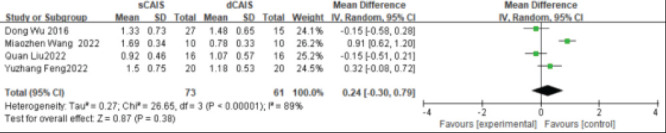
Forest plot of measurement deviation of root tip of implants

The measurement deviation of the root tip of the implant was included in 4 studies. The random effects model analysis showed that there was no statistically significant difference in the measurement deviation (mm) between the preoperative design and actual implantation site of the dynamic navigation and static guide plate groups at the root tip [WMD=0.24, 95% CI: (-0.30, 0.79), P=0.38] (Figure 6).

#### Measurement Deviation of Implant Angle

**Figure 7:**
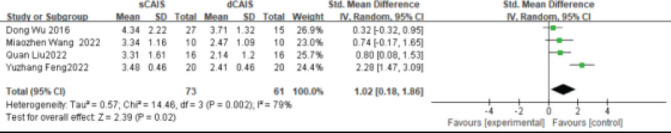
Forest plot of meta analysis results for measurement deviation of implant angle

The measurement deviation of implant angle was included in a total of 4 studies. Random effects model analysis showed that there was a significant difference in the measurement deviation (°) between the preoperative design and actual implant site angle between the dynamic navigation and static guide groups, with the dynamic navigation group having a smaller angle deviation ([WMD=1.01, 95%CI: (0.18, 1.86), P=0.02] (Figure 7).

## Discussion

The aesthetic area is the visible area when the patient smiles, generally referring to the anterior maxillary tooth area. Planting in this area involves many risks, on the one hand, patients have extremely high aesthetic requirements; On the other hand, tooth loss in the aesthetic area of the anterior teeth is often accompanied by absorption of alveolar bone, which seriously affects the placement of the implant^13^, especially the absorption of the labial bone plate of the anterior teeth; At the same time, the implantation surgery in the upper anterior teeth area should avoid special anatomical structures such as the incisor canal, to avoid causing damage to the nasopharyngeal nerve and affecting the bone union of the implant.

This review systematically evaluates the literature on the accuracy and clinical outcomes of static computer-assisted dental implantation. Scholars have conducted research on the accuracy of using static guide plates in different tooth positions, and found that the measurement deviation of the apical area, depth, and angle of the maxillary anterior teeth group are smaller than those of the maxillary first molar group^14^. However, there is currently no analysis of the accuracy deviation between the two navigation systems in the aesthetic area of the anterior teeth, Therefore, this study analyzes the importance of dynamic navigation and static guide plate implantation in the aesthetic area of anterior teeth implantation surgery.

Implantation in aesthetic areas presents specific risks and challenges primarily due to the high aesthetic demands and the potential impact of alveolar bone resorption. High aesthetic requirements mean that any minor misalignment or imperfection can be significantly more noticeable and less acceptable, especially in the front of the mouth where the teeth are most visible. This necessitates extremely precise placement and alignment of the implants.

Furthermore, alveolar bone resorption, which is the loss of bone around the teeth, can pose a challenge for implant placement. It can affect both the stability of the implant and the overall aesthetic outcome. The resorption may lead to insufficient bone volume, making it difficult to place the implant without additional procedures like bone grafting. This can complicate the surgery and extend the treatment time. Managing these risks requires meticulous planning and execution, often involving advanced imaging techniques and careful consideration of the patient's specific anatomical and aesthetic needs.

The smaller the number of missing teeth, the more anatomical markers can be used for navigation registration, and virtual design data can be more accurately converted into intraoperative^15^. In this study, there was no significant difference between the dynamic and static system and the static guide plate in terms of single tooth implantation. The results of this article show that there is no significant difference in the accuracy of preoperative design and actual implantation sites at the top and apex of the implant for implant surgery assisted by dynamic navigation and static guide plates. However, the dynamic navigation group has higher accuracy in angle deviation than the static guide plate group.

The accuracy of implant implantation will affect the later results of implant restoration. In the digital information age today, the diagnosis and treatment of implant restoration cannot be separated from the use of digital technology. Although digital guided implant surgery still cannot completely avoid implantation errors, it may have significant advantages over traditional implant methods such as shorter surgical time, less postoperative trauma, and higher accuracy. Currently, systematic reviews and meta-analyses have mostly focused on single center studies of dynamic navigation or static guides, or in vitro model studies comparing dynamic navigation and static guides^16^. The focus of this study is to compare and analyze the accuracy of dynamic navigation and static guide plates in aesthetic planting areas. The research results indicate that auxiliary applications of dynamic navigation and static guides can achieve similar accuracy.

Nowadays, with the popularity of digital guided technology, the application of digital implant guides in oral implant medicine makes it easy for doctors to perform surgeries regardless of whether they have clinical implant experience or not^17^. It simplifies the surgical steps, shortens the operating time, and enables the implantation surgery process to proceed in an orderly manner. However, compared to novices, experienced implant doctors are more familiar with surgical techniques, can predict surgical errors, and improve implant accuracy. Hamilton et al.^3^ believe that clinical experience has a limited impact on the accuracy of guide plate implantation, butstatistically speaking, clinical experience can reduce implant implantation errors and reduce the incidence of postoperative complications. Meanwhile, Schnutenhaus et al.^18^ viewed clinical experience as a potential factor affecting the accuracy of guide plate implantation. Siqueira et al.^19^ proposed that implant doctors need rich clinical experience and comprehensive knowledge to better use implant guides. Therefore, the surgeon should improve their clinical operating skills and cultivate a sense of touch during the operation, as the touch can provide clues to the location where the drill needle enters, and if necessary, direct observation can provide a more intuitive judgment and make necessary adjustments to the direction. The implant restoration technology using digital implant guides is an emerging technology that combines the restoration oriented and minimally invasive implant restoration concept with modern digital technology and 3D imaging, and is in line with the development trend of implant restoration.

By exploring the impact of static computer-assisted implant surgery on the accuracy of implant surgery in the aesthetic area of anterior teeth, we hope to have guiding significance for clinical work, in order to reduce the error of guide plate implant technology, improve implant accuracy, provide personalized treatment plans for patients with missing teeth, and achieve safe and accurate functional and aesthetic reconstruction. With the development and progress of science and technology, it is believed that there will be more advanced and Mature static guide plate planting technology applied to clinical practice to help reduce the error of implant surgery, ensure accuracy and standardization, and truly realize the implant repair treatment concept of restoration oriented, safe, accurate, efficient, minimally invasive, and aesthetic. In implant surgeries, clinical experience is a pivotal factor that significantly influences the accuracy and success of the procedure. Surgeons with extensive experience bring a depth of practical knowledge that aids in critical decision-making, especially in complex cases. Their refined skills and mastery of surgical techniques contribute to more accurate and precise implant placements. Experienced clinicians are adept at problem-solving, quickly identifying and addressing any unforeseen issues during the procedure. Moreover, they possess the adaptability to modify surgical plans in real-time, considering each patient's unique anatomical features. This combination of decision-making, skill, problem-solving, and adaptability, honed through years of clinical practice, is crucial for achieving optimal outcomes in implant surgeries, underscoring the indispensable value of experience in this field. The lack of homogeneity of research design in the publications included in the review limits this Systematic review. Many different surgical factors and techniques are not standardized between studies, which confuses the true accuracy of guided surgery. In addition, digital workflows themselves have many steps that may accumulate errors, which can also help conceal the true accuracy of the technology. Relying solely on radiological imaging techniques to compare pre treatment and post treatment positions is also considered another potential source of error, and future research should seek to use alternative comparative methods. In addition, few studies have focused on the value of guided surgery in achieving expected prosthetic plans or ultimate aesthetic outcomes. Although these limitations have been recognized, the trend in digital workflows is to improve accuracy. In addition, the author has decided to review only English publications, which may result in a lack of information published in other languages.

For future research to enhance the understanding of implant accuracy in aesthetic areas, it is essential to focus on several key aspects. Longitudinal studies are needed to evaluate the long-term stability and aesthetic outcomes of implants. Comparative studies should be conducted to assess the efficacy of different computer-assisted implant surgery technologies in aesthetic contexts. Additionally, exploring the impact of emerging technologies like augmented reality and AI on implant precision will be valuable. Understanding how individual patient factors affect implant success is crucial for personalized treatment approaches. Lastly, standardizing measurement techniques for implant placement accuracy will facilitate more consistent and comparable research findings across studies. This comprehensive approach will significantly contribute to advancements in implantology, particularly in aesthetic zones.

Meta regression analysis results of this study showed that there was no statistically significant difference in the accuracy of the implant area in the anterior aesthetic area under static guide and dynamic navigation, and there was no statistically significant difference in the impact of the measurement deviation of the implant area in the anterior aesthetic area under static guide and dynamic navigation. The angle deviation of the depth of the dynamic navigation group was smaller than that of the static guide group, and the difference was statistically significant. Considering the insufficient data and the errors caused by some uncontrollable variables, the conclusions of Meta regression analysis should be carefully considered. Overall, research has shown that although static navigation is greater than dynamic piracy in terms of angle deviation, there is currently no significant difference between static guide assisted oral implant implantation and dynamic navigation, which is still within the clinically acceptable range. The implant area is in the aesthetic area of the anterior teeth, and the study is designed as a model study or clinical study. However, due to the limited number of included studies, more high-quality studies are still needed to further analyze the influencing factors of the difference in implant accuracy.

## References

[1] Jorba-Garcia A, Gonzalez-Barnadas A, Camps-Font O, Figueiredo R, Valmaseda-Castellon E (2021). Accuracy assessment of dynamic computer-aided implant placement: a systematic review and meta-analysis. Clin Oral Invest.

[2] Chen P, Nikoyan L (2021). Guided Implant Surgery: A Technique Whose Time Has Come. Dent Clin North Am.

[3] Hamilton A, Singh A, Friedland B, Jamjoom FZ, Griseto N, Gallucci GO (2022). The impact of cone beam computer tomography field of view on the precision of digital intra-oral scan registration for static computer-assisted implant surgery: A CBCT analysis. Clin Oral Implan Res.

[4] Afrashtehfar KI (2021). Conventional free-hand, dynamic navigation and static guided implant surgery produce similar short-term patient-reported outcome measures and experiences. Evid Based Dent.

[5] Struwe M, Leontiev W, Connert T, Kuhl S, Filippi A, Herber V (2023). Accuracy of a dynamic navigation system for dental implantation with two different workflows and intraoral markers compared to static-guided implant surgery: An in-vitro study. Clin Oral Implan Res.

[6] Tahmaseb A, Wu V, Wismeijer D, Coucke W, Evans C (2018). The accuracy of static computer-aided implant surgery: A systematic review and meta-analysis. Clin Oral Implan Res.

[7] Yu X, Tao B, Wang F, Wu Y (2023). Accuracy assessment of dynamic navigation during implant placement: A systematic review and meta-analysis of clinical studies in the last 10 years. J Dent.

[8] Higgins JP, Altman DG, Gotzsche PC, Juni P, Moher D, Oxman AD (2011). The Cochrane Collaboration's tool for assessing risk of bias in randomised trials. BMJ-Brit Med J.

[9] Wang M, Rausch-Fan X, Zhan Y, Shen H, Liu F (2022). Comparison of Implant Placement Accuracy in Healed and Fresh Extraction Sockets between Static and Dynamic Computer-Assisted Implant Surgery Navigation Systems: A Model-Based Evaluation. Materials.

[10] Wu D, Zhou L, Yang J, Zhang B, Lin Y, Chen J (2020). Accuracy of dynamic navigation compared to static surgical guide for dental implant placement. Int J Implant Dent.

[11] Liu Q, Liu Y, Chen D, Wu X, Huang R, Liu R (2022). Placement accuracy and primary stability of implants in the esthetic zone using dynamic and static computer-assisted navigation: A retrospective case-control study. J Prosthet Dent.

[12] Feng Y, Su Z, Mo A, Yang X (2022). Comparison of the accuracy of immediate implant placement using static and dynamic computer-assisted implant system in the esthetic zone of the maxilla: a prospective study. Int J Implant Dent.

[13] Wang W, Zhuang M, Li S, Shen Y, Lan R, Wu Y (2023). Exploring training dental implant placement using static or dynamic devices among dental students. Eur J Dent Educ.

[14] Melo M, Ata-Ali F, Huertas J, Cobo T, Shibli JA, Galindo-Moreno P (2019). Revisiting the Maxillary Teeth in 384 Subjects Reveals A Deviation From the Classical Aesthetic Dimensions. Sci Rep-Uk.

[15] Yamaguchi K, Munakata M, Kataoka Y, Uesugi T, Shimoo Y (2022). Effects of missing teeth and nasal septal deviation on maxillary sinus volume: a pilot study. Int J Implant Dent.

[16] Pellegrino G, Ferri A, Del FM, Prati C, Gandolfi MG, Marchetti C (2021). Dynamic Navigation in Implant Dentistry: A Systematic Review and Meta-analysis. Int J Oral Max Impl.

[17] Raabe C, Schuetz TS, Chappuis V, Yilmaz B, Abou-Ayash S, Couso-Queiruga E (2023). Accuracy of keyless vs drill-key implant systems for static computer-assisted implant surgery using two guide-hole designs compared to freehand implant placement: an in vitro study. Int J Implant Dent.

[18] Schnutenhaus S, Edelmann C, Knipper A, Luthardt RG (2021). Accuracy of Dynamic Computer-Assisted Implant Placement: A Systematic Review and Meta-Analysis of Clinical and In Vitro Studies. J Clin Med.

[19] Siqueira R, Chen Z, Galli M, Saleh I, Wang HL, Chan HL (2020). Does a fully digital workflow improve the accuracy of computer-assisted implant surgery in partially edentulous patients? A systematic review of clinical trials. Clin Implant Dent R.

